# Synthesis of New *C_2_-*Symmetric Fluoren-9-ylidene Malonate Derived Bis(oxazoline) Ligands and Their Application in Friedel-Crafts Reactions

**DOI:** 10.3390/molecules15128582

**Published:** 2010-11-26

**Authors:** Jing Li, Hong-Liang Chen, Lei Liu, Bin Fu

**Affiliations:** Department of Applied Chemistry, China Agricultural University, Beijing 100193, China; E-Mails: lijing_19901210@126.com (J.L.); chenhongliang1016@yahoo.cn (H.-L.C.); LiuLei-111@163.com (L.L.)

**Keywords:** bis(oxazoline), fluoren-9-ylidene, asymmetric catalysis, Friedel-Crafts

## Abstract

A series of new *C_2_*-symmetric fluoren-9-ylidene malonate-derived bis(oxazoline) ligands were synthesized from fluoren-9-ylidene malonate and enantiomerically pure amino alcohols *via* a convenient route. Their asymmetric catalytic properties in the Friedel-Crafts reactions of indoles with arylidene malonates were evaluated, and the Cu(OTf)_2_ complex of a fluoren-9-ylidene malonate-derived bis(oxazoline) bearing a phenyl group showed moderate to good enantioselectivity (up to 88% ee).

## 1. Introduction

**Figure 1:**
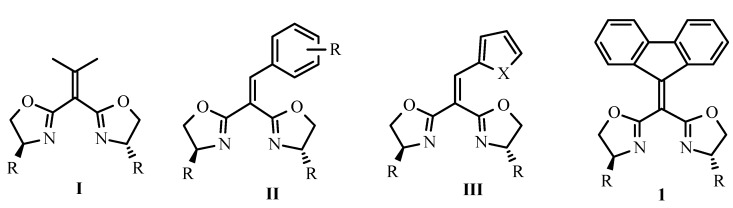
Typical alkylidene and arylidene malonate-type bis(oxazoline) ligands.

During the past two decades, a plethora of bis(oxazoline) ligands have been synthesized and successfully applied in a variety of asymmetric catalytic reactions [1,2,3]. For bis(oxazoline) (BOX) ligands derived from malonate and its analogues, the bridge angle φ, correlating with the bite angle θ of the BOX-metal complex, is an important structural factor influencing the enantioselectivity of the catalysis [4,5,6]. In recent years, one straightforward strategy to tune the bridge angle was introduced to BOX ligands, in which two oxazoline rings are attached to a sp^2^ hybridized carbon and then provide a larger bridge angle than those with sp^3^ hybridized bridge carbon. So far several examples involving this type of BOX ligand have appeared, such as **I** and **II** (Figure 1) [7,8,9]. Recently, we reported that heteroarylidene malonate-derived bis(oxazoline) ligand **III**-copper(II) complexes demonstrated excellent enantioselectivities (up to >99% ee) in the catalytic Friedel-Crafts reactions between indoles and diethyl alkylidenemalonates [10]. As a continuation of our ongoing endeavor to explore these novel chiral ligands and their application in synthetic methodology, herein we wish to document the synthesis and application of fluoren-9-ylidene malonate derived bis(oxazoline) **1.**

## 2. Results and Discussion

**Scheme 1:**
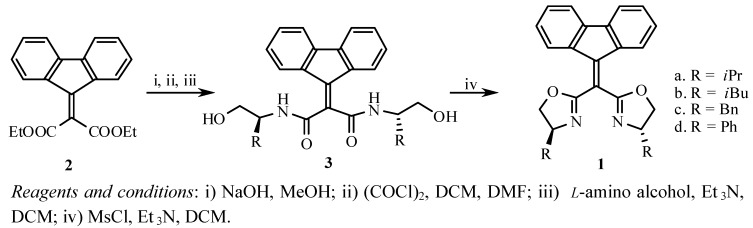
Synthesis of chiral bis(oxazolines) **1**.

The requisite chiral bis(oxazolines) **1** were conveniently synthesized from the commercially available starting material diethyl fluoren-9-ylidene malonate (**2**) in a four step sequence as illustrated in Scheme 1 [10]. Hydrolysis of diethyl dicarboxylates **2** by the solution of NaOH in a mixture of water and methanol gave the corresponding dicarboxylic acid, which reacted with oxalyl chloride in the presence of DMF to afford the diacyl chloride. The diacyl chloride condensed with chiral β-amino alcohols in the presence of Et_3_N to give the corresponding chiral intermediate dihydroxydiamides **3** in good yields, which were treated with methanesulfonyl chloride and excess Et_3_N in dichloromethane to afford the desired bis(oxazoline)s **1a**~**d** in good yield (75-84%). 

**Scheme 2:**
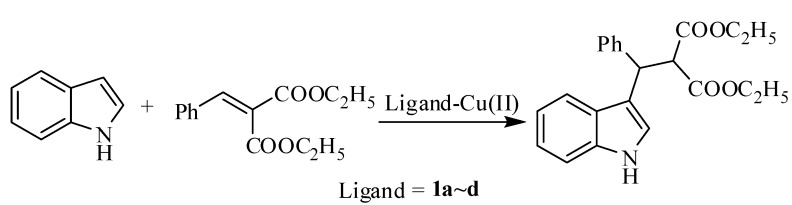
Friedel-Crafts alkylations catalyzed by Cu(OTf)_2_-bis(oxazoline) complexes **1a~d**.

With these new ligands in hand, we evaluated their catalytic activity in the Cu(II) catalyzed Friedel-Crafts (F-C) alkylation of indole with arylidene malonates according to our previous reports (Scheme 2) [10]. The asymmetric Friedel-Crafts alkylation of indoles with arylidene malonates affords an efficient methodology to prepare indole derivatives [11,12,13,14,15,16,17]. The F-C alkylation was performed in iso-butanol at room temperature employing Cu(OTf)_2_-bis(oxazoline) complexes **1a~d** (10 mol%) as catalysts (Scheme 2). The experimental results are outlined in Table 1. Ligand **1d** showed the best enantioselectivity (78% ee) among the four Cu(OTf)_2_-ligand complexes, while **1a~c** gave low catalytic enantioselectivities (entries 1, 2 and 3). When Cu(ClO_4_)_2_6H_2_O was used in this teaction, the ee was decreased to 34% (entry 5). Subsequently, the effect of solvents were examined. In isopropanol or ethanol, almost the same catalytic activities and enantioselectivities were exhibited (entries 6 and 7), however in methanol the enantioselectivity was reduced to 40% ee (Entry 8). When dichloromethane was used in this reaction, both a high yield and low enantioselectivity were obtained (90% yield and 20% ee, entry 9), which was not in accordance with our previous report that the use of dichloromethane as solvent led to the product with the the opposite configuration being obtained [10,15].

**Table 1:** Effect of ligands and solvent in Cu(II)-catalyzed Friedel-Crafts alkylations. ^a^

Entry	**Ligands**	Solvent	Salt	Yield (%)^b^	Ee (%)^c^
1	**1a**	isobutanol	Cu(OTf)_2_	99	39
2	**1b**	isobutanol	Cu(OTf)_2_	99	16
3	**1c**	isobutanol	Cu(OTf)_2_	99	57
4	**1d**	isobutanol	Cu(OTf)_2_	99	78
5	**1d**	isobutanol	Cu(ClO_4_)_2_H_2_O	99	34
6	**1d**	isopropanol	Cu(OTf)_2_	99	77
7	**1d**	ethanol	Cu(OTf)_2_	99	78.
8	**1d**	methanol	Cu(OTf)_2_	99	40
9	**1d**	CH_2_Cl_2_	Cu(OTf)_2_	90	25

^a^ All the reactions were conducted under nitrogen for 24 h using 10 mol% of catalyst at room temperature; ^b^ Isolated yield. ^c^ Determined by chiral HPLC.

Next the reactions of various indoles and alkylidene malonates were investigated under the optimal reaction conditions (Table 1, Entry 4). As shown in Table 2, different reactivities were observed for different substrates. The benzylidene malonates with *ortho*-ClPh and *ortho*-MePh groups afforded much lower yields after reacting 48 h (60% and 65%, respectively). The enantioselectivity of the reactions was found to depend significantly on the different substituents on the substrates (entries 1~5). The best result was achieved (up to 88% ee) when diethyl *ortho-*Cl-benzylidene malonate reacted with indole (Entry 4). On the other hand, in the reaction of various substituted indoles with diethyl benzylidene malonates, inferior enantioselectivities (10~45% ee, entries 6~9) resulted for the adducts of the benzylidene malonate reacting with 5-methoxyindole, 5-methylindole, 5-chloroindole and 6-chloroindole, although high yields were obtained.

**Scheme 3:**
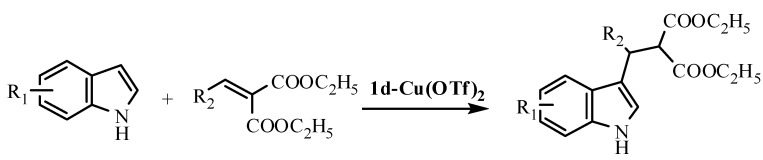
F-C alkylations of different indoles and malonates.

**Table 2:** 1d-Cu(OTf)_2_ catalyzed Friedel-Crafts reaction of indole derivatives with alkylidene malonates. ^a^

Entry	R^1^	R^2^	Time (h)	Yield (%)^b^	ee^c^ (%)	Config.
1	H	*p*-MeC_6_H_4_	24	99	37	*S*
2	H	*p*-FC_6_H_4_	24	90	31	*S*
3	H	*m*-BrC_6_H_4_	48	95	52	*S*
4	H	*o*-ClC_6_H_4_	48	60	88	*S*
5	H	*o-*MeC_6_H_4_	48	65	15	*S*
6	5-MeO	C_6_H_5_	24	99	41	*S*
7	5-Me	C_6_H_5_	24	99	47	*S*
8	5-Cl	C_6_H_5_	48	90	45	*S*
9	6-Cl	C_6_H_5_	48	80	10	*S*

*^a^* All reactions were conducted in isobutanol under nitrogen using 10 mol% catalyst at room temperature; *^b^* Isolated yield; *^c^* Determined by chiral HPLC.

## 3. Experimental

### 3.1. General

Melting points were measured on an XT-4 melting point apparatus and are uncorrected. NMR spectra were recorded with a Bruker Avance DPX300 spectrometer with tetramethylsilane as the internal standard. Infrared spectra were obtained on a Nicolet AVATAR 330 FT-IR spectrometer; Optical rotations were measured on a Perkin–Elmer 341 LC polarimeter. Elemental analyses were carried out on an Elementar Vario EL instrument. The enantiomeric excesses of (*R*)- and (*S*)-ethyl-2-ethoxycarbonyl-3-(3-indolyl)-3-arylpropanoate were determined by HPLC analysis over a chiral column (Daicel Chiralcel OD-H; *n*-hexane/*i*-PrOH 90:10, 0.8 mL/min; UV detector, 254 nm). The absolute configuration of the major enantiomer was assigned by comparison with literature [10,15].

### 3.2. Synthesis and characterization of dihydroxydiamides ***3a-d***

#### 3.2.1. (*S,S*)-*N,N*-bis(2-hydroxy-1-isopropyl)-2-(fluoren-9-ylidene) malonamide (**3a**)

To a solution of diethyl fluoren-9-ylidene malonate **2a** (1.0 g, 3.10 mmol) in CH_3_OH (10 mL) was added a NaOH solution (10 mL, 2.0 M). The mixture was refluxed for 8 h, then the methanol was removed *invacuo*. The residue was cooled to 0 °C and acidified with aqueous HCl (6 M). The acidified mixture was extracted with ethyl acetate (10 mL × 3), and the combined organic phase was washed with brine, dried over Na_2_SO_4_ and evaporated to give yellow solid, which was directly added to a solution of CH_2_Cl_2_ (20 mL) and DMF (0.1 mL), subsequently at 0 °C oxalyl chloride (1.20 g, 9.44 mmol) was slowly injected and then the mixture stirred for 3 h. Removal of the excess oxalyl chloride *in vacuo* afforded the diacyl dichloride as a yellow solid. The diacyl dichloride in CH_2_Cl_2_ (20 mL) was added dropwise to a solution of L-valinol (0.75 g, 7.28 mmol) and Et_3_N (4 mL, 28.9 mmol) in CH_2_Cl_2_ (20 mL) at 0 °C and stirred at room temperature for 4 h. The reaction mixture was washed with water (5 mL × 2). The organic layer was dried over Na_2_SO_4_ and concentrated to give crude solid. Purification by silica gel column chromatography (70% ethyl acetate in petroleum ether) afforded the dihydroxydiamide **3a**. Yield: 1.16 g (86%) as a yellow solid. m.p. 238.0~239.5 °C; [α]_D_^25^ = +66.0 (c = 0.10, CH_2_Cl_2_). IR (cm^-1^): 3252, 3061, 2962, 1633, 1540, 1449, 1317, 1057, 725; ^1^H-NMR (DMSO): *δ* 8.22 (d, *J* = 8.88 Hz, 2H, NH), 7.83 (dd, *J* = 7.50, 11.70 Hz, 4H, ArH), 7.43-7.38 (m, 2H, ArH), 7.27-7.22 (m, 2H, ArH), 4.60(s, 2H, OH), 3.89-3.85 (m, 2H, C*H*NH), 3.54-3.41 (m, 4H, C*H*_2_O), 1.99-1.93 (m, 2H, C*H*Me_2_), 0.91 (d, *J* = 6.87 Hz, 6H, CH_3_), 0.85 (d, *J* = 6.87 Hz, 6H, CH_3_); ^13^C-NMR (DMSO): *δ* 165.2, 139.9, 135.7, 134.5, 131.7, 129.5, 127.5, 124.9, 120.0, 61.1, 56.1, 28.3, 19.9, 17.6; Anal. Calcd. for C_26_H_32_N_2_O_4_ (436.55): C 71.53, H 7.39, N 6.42; Found: C 71.79, H 7.65, N 6.33.

#### 3.2.2. (*S,S*)-*N,N*-bis(2-hydroxy-1-isobutyl)-2-(fluoren-9-ylidene) malonamide (**3b**)

Prepared according to procedure 3.2.1. Yield: 1.20 g (84%). m.p. 223~224.5 °C; [α]_D_^25^ = +84.4 (c = 0.15, CH_2_Cl_2_); IR (cm^-1^): 3254, 3060, 2956, 2870, 1638, 1542, 1449, 1385, 1367, 1320, 1064, 774, 725; ^1^H-NMR (DMSO): *δ* 8.21(d, *J* = 11.70 Hz, 2H, NH), 7.84 (dd, *J* = 7.35, 12.60 Hz, 4H, ArH), 7.43-7.38 (m, 2H, ArH), 7.26-7.21 (m, 2H, ArH), 4.81(s, 2H, OH), 4.05-4.04 (m, 2H, C*H*NH), 3.50 (dd, *J* = 5.10, 10.20 Hz, 2H, CH_2_O), 3.35-3.27(m, 2H, CH_2_O), 1.70-1.61(m, 2H, C*H*CH_2_), 1.43-1.29 (m, 4H, CH_2_), 0.92 (dd, *J* = 6.48 Hz, 6H, CH_3_), 0.88 (dd, *J* = 6.60 Hz, 6H, CH_3_); ^13^C-NMR (DMSO): *δ* 164.9, 139.9, 135.7, 134.4, 131.8, 129.5, 127.3, 124.8, 120.1, 63.7, 49.3, 24.1, 23.8, 21.8; Anal. Calcd. for C_28_H_36_N_2_O_4_ (464.60): C 72.39, H 7.81, N 6.03; Found: C 72.54, H 7.68, N 6.31.

#### 3.2.3. (*S,S*)-*N,N*-bis(2-hydroxy-1-benzyl)-2-(fluoren-9-ylidene) malonamide (**3c**)

Prepared according to procedure 3.2.1. Yield: 1.45 g (88 %). m.p. 197.5~198.5 °C; [α]_D_^25^ = +23.0 (*c* = 0.45, CH_2_Cl_2_). IR (cm^-1^): 3423, 1637, 1627, 1537, 1449, 1035, 775, 727, 701; ^1^H-NMR (DMSO): *δ* 8.43-8.41(d, *J* = 8.40 Hz, 2H, NH), 7.78 (d, *J* = 7.50 Hz, 2H, ArH), 7.48-7.45(d, *J* = 7.80 Hz, 2H, ArH), 7.37-7.17(m, 10H, ArH), 7.05(t, *J* = 7.50 Hz, 2H, ArH), 4.92 (t, *J* = 5.25 Hz, 2H), 4.24-4.19 (m, 2H, C*H*NH), 3.52-3.45 (m, 2H, C*H*_2_O), 3.42-3.35(m, 2H, C*H*_2_O), 2.94 (dd, *J* = 6.09, 13.50 Hz, 2H, C*H*_2_Ph), 2.74 (dd, *J* = 7.80, 13.80 Hz, 2H, C*H*_2_Ph); ^13^C-NMR (DMSO): *δ* 164.8, 139.9, 139.0, 135.5, 133.8, 132.5, 129.4, 129.3, 128.4, 127.5, 126.3, 124.7, 119.9, 62.2, 53.0, 36.6; Anal. Calcd. for C_34_H_32_N_2_O_4_ (532.63): C 76.67, H 6.06, N 5.26; Found: C 76.62, H 6.20, N 5.37.

#### 3.2.4. (*S,S*)-*N,N*-bis(2-hydroxy-1-phenyl)-2-(fluoren-9-ylidene) malonamide (**3d**)

Prepared according to procedure 3.2.1. Yield: 1.33g (85%). m.p. 238~239 °C; [α]_D_^25^ = +40.0 (*c* = 0.1, CH_2_Cl_2_). IR (cm^-1^): 3436, 1639, 1532, 1449, 1040, 720, 700; ^1^H-NMR (DMSO): *δ* 9.05 (d, *J* = 8.10 Hz, 2H, NH), 7.81 (d, *J* = 7.50 Hz, 2H, ArH), 7.45-7.30 (m, 12H, ArH), 7.01-6.96 (m, 2H, ArH), 5.08 (dd, *J* = 7.50, 13.50 Hz, 2H, C*H*NH), 5.02 (t, *J* = 5.10 Hz, 2H, OH), 3.71-3.65 (m, 4H, CH_2_O); ^13^C-NMR (DMSO): *δ* 164.5, 140.3, 139.9, 135.4, 133.6, 132.6, 129.5, 128.3, 127.4, 127.4, 127.2, 124.8, 119.9, 64.5, 55.6; Anal. Calcd. for C_32_H_28_N_2_O_4_ (504.58): C 76.17, H 5.59, N 5.55; Found: C 76.01, H 5.70, N 5.27.

### 3.3. The synthesis and characterization of bis(oxazoline) ligands ***1a-d***

#### 3.3.1. Bis[(*S*)-4-iso-propyloxazoline-2-yl]-2-(fluoren-9-yl)-ethene (**1a**)

MsCl (0.30 g, 2.63 mmol) was slowly added to an ice-cooled solution of the dihydroxydiamide **3a** (0.50 g, 1.15 mmol) and Et_3_N (4 mL, 28 mmol) in CH_2_Cl_2_ (20 mL). The mixture was allowed to warm to room temperature and stirred for 12 h. The mixture was washed with water (2 × 5 mL). The organic layer was dried over anhydrous Na_2_SO_4_ and concentrated to dryness*invacuo*, the residue was purified by flash chromatography on silica gel (ethyl acetate/petroleum ether, 1/1, v/v) to afford **1a** as a yellow solid. Yield: 0.36 g (78%); m.p. 163~164.5 °C; [α]_D_^25^ = -88.6 (c = 0.50, CH_2_Cl_2_). IR (cm^-1^): 2956, 2874, 1652, 1609, 1481, 1447, 1370, 1016, 946, 785, 732; ^1^H-NMR (CDCl_3_): *δ* 7.73 (d, *J* = 7.46 Hz, 2H), 7.58 (d, *J* = 7.50Hz, 2H), 7.35-7.30 (m, 2H, ArH), 7.21-7.15 (m, 2H, ArH), 4.51-4.46 (m, 2H, C*H*N=), 4.26-4.15 (m, 4H, CH_2_O), 1.96 (t, *J* = 6.63 Hz, 2H, C*H*Me), 1.06 (d, *J* = 6.75 Hz, 6H, CH_3_), 0.99 (d, *J* = 6.75 Hz, 6H, CH_3_); ^13^C-NMR (CDCl_3_): *δ* 161.3, 144.8, 141.5, 136.5, 130.2, 127.2, 125.9, 119.5, 115.1, 73.4, 70.3, 32.7, 19.1, 18.3; Anal. Calcd. for C_26_H_28_N_2_O_2_ (400.51): C 77.97, H 7.05, N 6.99; Found: C 77.98, H 7.24, N 7.07.

#### 3.3.2. Bis[(*S*)-4-iso-butyloxazoline-2-yl]-2-(fluoren-9-yl)-ethene (**1b**)

Prepared according to procedure 3.3.1, starting from **3b** (0.5 g, 1.08 mmol) and MsCl (0.28 g, 2.46 mmol) in CH_2_Cl_2_ (15.0 mL); yellow solid; yield: 0.39 g (84 %); m.p. 67.0~68.5 °C; [α]_D_^25^ = -88.8 (*c* = 0.25, CH_2_Cl_2_). IR (cm^-1^): 2955, 1648, 1468, 1449, 1368, 1276, 1152, 1040, 1003, 938, 786, 728; ^1^H-NMR (CDCl_3_): *δ* 7.75 (d, *J* = 7.80 Hz, 2H, ArH), 7.58 (d, *J* = 7.50 Hz, 2H, ArH), 7.36-7.30 (m, 2H, ArH), 7.21-7.16 (m, 2H, ArH), 4.59-4.53 (dd, *J* = 7.80, 9.60 Hz, 2H, C*H*_2_O), 4.48-4.40 (m, 2H, C*H*N=), 4.05 (t, *J* = 7.80 Hz, 2H, C*H*_2_O), 1.90-1.78 (m, 4H, CH_2_), 1.52-1.41 (m, 2H, C*H*Me_2_), 0.99 (t, *J* = 6.0 Hz, 12H, CH_3_); ^13^C-NMR (CDCl_3_): *δ* 161.1, 144.8, 141.5, 136.4, 130.2, 127.1, 125.9, 119.4, 114.9, 73.2, 70.3, 65.6, 44.9, 25.3, 22.6, 22.6; Anal. Calcd. for C_28_H_32_N_2_O_2_ (428.57): C 78.47, H 7.53, N 6.54. Found: C 78.66, H 7.75, N 6.57.

#### 3.3.3. Bis[(*S*)-4-benzyloxazoline-2-yl]-2-(fluoren-9-yl)-ethene (**1c**)

Prepared according to the procedure 3.3.1, starting from **3c** (0.50 g, 0.94 mmol) and MsCl (0.25 g, 2.19 mmol) in CH_2_Cl_2_ (15.0 mL); yellow solid; yield: 0.36g (77%); m.p. 120.0~122.0 °C; [α]_D_^25^ = -108.8 (*c* = 0.25, CH_2_Cl_2_). IR (cm^-1^): 2957,1649, 1614, 1496, 1449, 1309, 1228, 1147, 1018, 977, 783, 728, 700; ^1^H-NMR (CDCl_3_): *δ* 7.61 (d, *J* = 7.84 Hz, 2H), 7.56 (d, *J* = 7.23 Hz, 2H, ArH), 7.35-7.21 (m, 12H, ArH), 7.16-7.11(m, 2H), 4.79-4.68 (m, 4H, C*H*N=), 4.45(t, *J* = 8.74 Hz, 2H, C*H*_2_O), 4.18 (t, *J* = 8.44 Hz, 2H, C*H*_2_O), 3.32 (dd, *J* = 5.18, 13.81Hz, 2H, C*H*_2_Ph), 2.90 (dd, *J* = 8.60, 13.80 Hz, 2H, C*H*_2_Ph); ^13^C-NMR (CDCl_3_): *δ* 162.0, 145.4, 141.6, 137.6, 136.4, 130.4, 129.4, 128.6, 127.3, 126.6, 126.0, 119.5, 114.4, 72.0, 68.5, 41.1; Anal. Calcd. for C_34_H_28_N_2_O_2_ (496.60): C 82.23, H 5.68, N 5.64. Found: C 82.47, H 5.77, N 5.47.

#### 3.3.4. Bis[(*S*)-4-phenyloxazoline-2-yl]-2-(fluoren-9-yl)-ethene (**1d**)

Prepared according to the procedure 3.3.1, starting from **3d** (0.50 g, 0.99 mmol) and MsCl (0.25 g, 2.19 mmol) in CH_2_Cl_2_ (15.0 mL); yellow solid; yield: 0.35 g (75 %); m.p. 167.5~168.5 °C; [α]_D_^25^ = -104.4 (*c* = 0.45, CH_2_Cl_2_); IR (cm^-1^): 1653, 1448, 1356, 1268, 1204, 1145, 1017, 957, 938, 786, 735, 701; ^1^H-NMR (CDCl_3_): *δ* 7.79 (d, *J* = 7.83 Hz, 2H, ArH), 7.60 (d, *J* = 7.47 Hz, 2H, ArH), 7.42-7.26 (m, 12H, ArH), 7.18-7.15(m, 2H, ArH), 5.57 (dd, *J* = 8.64, 10.26 Hz, 2H, C*H*N=), 4.90 (dd, *J* = 8.61, 10.32 Hz, 2H, CH_2_O), 4.38 (t, *J* = 8.61 Hz, 2H, CH_2_O); ^13^C-NMR (CDCl_3_): *δ* 162.9, 146.1, 141.7, 141.6, 136.40, 130.6, 128.8, 127.7, 127.4, 126.9, 126.2, 119.6, 114.0, 74.8, 70.7; Anal. Calcd. for C_32_H_24_N_2_O_2_ (468.55): C 82.03, H 5.16, N 5.98; Found: C 82.22, H 5.27, N 5.89.

### 3.4. General procedure for the asymmetric F-C alkylation of indoles with alkylidenemalonates

Cu(OTf)_2_ (0.025 mmol) was added to a Schlenk tube, followed by ligand **1d** (0.0275 mmol) in iso*-*butanol (1.0 mL) under N_2_, the solution was stirred for 1.5 h at room temperature, a mixture of the appropriate diethyl arylidenemalonate (0.25 mmol) in the above solvent (1.0 mL) was added. After stirring for 30 min the indole (0.25 mmol) was added. After stirring for 24~48 h at room temperature, the solution was concentrated *in vacuo*, The crude product was purified by flash column chromatography on silica gel (eluted with ethyl acetate-petroleum ether, 1/5, v/v) to afford the (*S*)-ethyl-2-ethoxycarbonyl-3-(3-indolyl)-3-arylpropanoate as a white solid in high yield; the enantiomeric excesses of all adducts were determined by HPLC with a chiral column (Daicel Chiralcel OD-H; hexane-isopropyl alcohol 90:10; flow rate 0.8 mL/min; 254 nm). 

#### 3.4.1. (*S*)–Ethyl 2–ethoxycarbonyl–3–(3–indolyl)–3–phenyl propanoate

Prepared according to the general procedure from diethyl benzylidenemalonate and indole to provide the pure product as a white solid; m.p. 172-176 °C; [α]_D_^25^ = + 33.6 (*c* = 0.45, CH_2_Cl_2_); ^1^H-NMR (CDCl_3_): *δ* 8.04 (brs, 1H, NH), 7.55 (d, *J* = 8.0 Hz, 1H, ArH), 7.09-7.37 (m, 8H, ArH), 7.00-7.07(m, 1H, ArH), 5.07 (d, *J* = 11.7 Hz, 1H, CH), 4.28 (d, *J* = 11.7 Hz, 1H, CH), 3.93-4.04 (m, 4H, OCH_2_), 0.96-1.03 (m, 6H, CH_3_); ^13^C-NMR (CDCl_3_): *δ* 168.1, 167.9, 141.4, 136.2, 128.4, 128.2, 126.8, 126.7, 122.3, 120.9, 119.5, 119.4, 117.0, 111.0, 61.5, 61.4, 58.4, 42.9, 13.8. HPLC analysis: t_r_ (minor) = 12.43 min, t_r_ (major) = 15.14 min, 78% ee.

#### 3.4.2. (*S*)–Ethyl 2–ethoxycarbonyl–3–(3–indolyl)–3–(*p*-methylphenyl) propanoate

Prepared according to the general procedure from diethyl *p*-methylbenzylidenemalonate and indole to provide the pure product as a white solid; m.p. 137-139 °C; [α]_D_^25^ = +10.5 (*c* = 0.50, CH_2_Cl_2_); ^1^H-NMR (CDCl_3_): *δ* 7.97 (s, 1H, NH), 7.54 (d, *J* = 7.80 Hz, 1H, ArH), 7.29-7.23 (m, 3H, ArH), 7.16-7.09 (m, 2H, ArH), 7.04-6.99 (m, 2H, ArH), 5.03 (d, *J* = 11.70 Hz, 1H, CH), 4.26 (d, *J* = 11.70 Hz, 1H, CH), 2.24 (s, 3H, CH_3_), 1.03 (t, *J* = 6.90 Hz, 3H, CH_3_), 0.97 (t, *J* = 6.90 Hz, 3H, CH_3_). ^13^C-NMR (CDCl_3_): *δ* 168.1, 167.9, 138.4, 136.2, 136.2, 129.0, 128.0, 126.7, 122.2, 120.8, 119.5, 119.5, 117.3, 110.9, 61.4, 61.4, 58.4, 42.4, 21.0, 13.8. HPLC analysis: t_r_ (minor) = 14.99 min, t_r_ (major) = 16.28 min, 37% ee.

#### 3.4.3. (*S*)–Ethyl 2–ethoxycarbonyl–3–(3–indolyl)–3–(*p*-fluorophenyl)) propanoate

Prepared according to the general procedure from diethyl *p*-fluorobenzylidenemalonate and indole to provide the pure product as a white solid; m.p. 132.5-134 °C; [α]_D_^25^ = +22.0 (*c* = 0.50, CH_2_Cl_2_); ^1^H-NMR (CDCl_3_): *δ* 8.03 (s, 1H, NH), 7.62 (d, *J* = 8.10 Hz, 1H, ArH), 7.32-7.05 (m, 5H, ArH), 6.98 (d, *J* = 2.76 Hz, 1H, ArH), 6.84 (dd, *J* = 3.90, 5.10 Hz, 1H, ArH), 5.38 (d, *J* = 11.40 Hz, 1H, CH), 4.29 (d, *J* = 11.40 Hz, 1H, CH), 4.10(q, *J* = 7.20 Hz, 2H, OCH_2_), 3.93 (q, *J* = 6.99 Hz, 2H, OCH_2_), 1.13 (t, *J* = 6.84 Hz, 3H, CH_3_), 0.91 (t, *J* = 7.08 Hz, 3H, CH_3_); ^13^C-NMR (CDCl_3_): *δ* 168.0, 167.8, 161.7, 137.5, 137.6, 136.4, 129.9, 129.8, 126.6, 122.5, 120.9, 119.8, 119.4, 116.9, 115.5, 115.2, 111.2, 61.8, 61.7, 58.5, 42.5, 13.9, 13.8. HPLC analysis: t_r_ (minor) = 15.57 min, t_r_ (major) = 18.19 min, 31% ee.

#### 3.4.4. (*S*)–Ethyl 2–ethoxycarbonyl–3–(3–indolyl)–3–(*m*-bromophenyl) propanoate

Prepared according to the general procedure from diethyl *m*-bromobenzylidenemalonate and indole to provide the pure product as a white solid; m.p. 123-124 °C; [α]_D_^25^ = +24.0 (*c* = 0.20, CH_2_Cl_2_); ^1^H-NMR (CDCl_3_): *δ* 8.05(s, 1H, NH), 7.50 (dd, 2H, ArH), 7.32-7.02 (m, 7H, ArH), 5.04 (d, *J* = 12.0 Hz, 1H, CH),4.23 (d, *J* = 12.0 Hz, 1H), 4.05-3.98 (m, 4H, OCH_2_), 1.08-0.96 (m, 6H, CH_3_); ^13^C-NMR (CDCl_3_): *δ* 167.8, 167.7, 146.9, 146.3, 131.3, 129.9, 127.0, 126.5, 122.4, 122.4, 121.1, 119.6, 119.1, 116.1, 111.2, 61.7, 58.2, 42.4, 13.9, 13.7. HPLC analysis: t_r_(minor) = 15.31 min, t_r_ (major) = 20.64 min), 52% ee.

#### 3.4.5. (*S*)–Ethyl 2–ethoxycarbonyl–3–(3–indolyl)–3–(*o*-chlorophenyl) propanoate

Prepared according to the general procedure from diethyl *o*-chlorobenzylidenemalonate and indole to provide the pure product as a white solid; m.p. 125-127 °C; [α]_D_^25^ = +22.6 (*c* = 0.50, CH_2_Cl_2_); ^1^H-NMR (CDCl_3_): *δ* 8.15 (s, 1H, NH), 7.68 (d, *J* = 7.80 Hz, 1H, ArH), 7.40-7.23(m, 3H, ArH), 7.14-7.04 (m, 5H, ArH), 5.66 (d, *J* = 11.70 Hz, 1H, CH), 4.40 (d, *J* = 11.70 Hz, 1H, CH), 4.04-3.92 (m, 4H, OCH_2_), 1.01 (t, *J* = 6.90 Hz, 3H, CH_3_), 0.94 (t, *J* = 6.90 Hz, 3H, CH_3_); ^13^C-NMR (CDCl_3_): *δ* 168.1, 167.7, 139.3, 136.2, 134.2, 129.9, 129.0, 128.1, 126.9, 126.8, 122.3, 122.2, 119.6, 119.7, 115.8, 111.3, 61.7, 57.8, 38.9, 13.8, 13.7. HPLC analysis: t_r_ (minor) = 15.88 min, t_r_ (major) = 20.63 min, 88% ee.

#### 3.4.6. (*S*)–Ethyl 2–ethoxycarbonyl–3–(3–indolyl)–3–(*o*-methylphenyl) propanoate

Prepared according to the general procedure from diethyl *o*-methylbenzylidenemalonate and indole to provide the pure product as a white solid; m.p. 94-95 °C; [α]_D_^25^ = +1.5 (*c* = 0.20, CH_2_Cl_2_). ^1^H-NMR (CDCl_3_): *δ* 7.87 (s, 1H, NH), 7.82 (d, *J* = 7.50 Hz, 2H, ArH), 7.64-7.62 (m, 1H, ArH), 7.37-7.25 (m, 3H), 7.20-7.00 (m, 3H, ArH), 5.31(d, *J* = 12.0 Hz, 1H, CH), 4.33(d, *J* = 12.0 Hz, 1H, CH), 4.00-3.86 (m, 4H, OCH_2_), 1.01-0.85 (m, 6H, CH_3_); ^13^C-NMR (75MHz, CDCl_3_): *δ* 168.4, 167.9, 140.1, 136.3, 135.9, 130.7, 126.8, 126.4, 126.3, 126.0, 122.3, 122.1, 119.5, 119.3, 116.5, 110.9, 61.4, 61.3, 58.5, 38.0, 19.9, 13.7, 13.6. HPLC analysis: t_r_ (minor) = 12.21 min, t_r_ (major) = 15.39 min, 15% ee.

#### 3.4.7. (S)-Ethyl 2-ethoxycarbonyl-3-[3-(5-methoxyindolyl)]-3-phenylpropanoate

Prepared according to the general procedure from diethyl benzylidenemalonate and 5-methoxyindole to provide the pure product as a white solid; m.p. 143-145 °C; [α]_D_^25^ = +6.0 (*c* = 0.20, CH_2_Cl_2_); ^1^H-NMR (CDCl_3_): *δ* 7.91(s, 1H, NH), 7.38-7.34 (m, 2H, ArH), 7.25-7.11 (m, 4H, ArH), 6.96 (d, *J* = 2.40Hz, 1H, ArH), 6.78 (dd, *J* = 2.40 Hz, 9.0 Hz, ArH), 5.01 (d, *J* = 12.0 Hz, CH), 4.25 (d, *J* = 12.0 Hz, CH), 4.05-3.94 (m, 4H, 2×CH_2_), 3.78 (s, 3H, OCH_3_), 1.00 (t, *J* = 7.20 Hz, 6H, 2×CH_3_). ^13^C-NMR (CDCl_3_): *δ* 168.1, 167.6, 140.8, 139.6, 131.5, 128.7, 127.4, 127.3, 126.7, 120.8, 111.7, 101.3, 61.6, 61.5, 58.4, 55.8, 42.5, 13.8. HPLC analysis: t_r_ (minor) = 20.40 min, t_r_ (major) = 27.86 min, 41% ee.

#### 3.4.8. (S)-Ethyl 2-ethoxycarbonyl-3-[3-(5-methylindolyl)]-3-phenylpropanoate

Prepared according to the general procedure from diethyl benzylidenemalonate and 5-methylindole to provide the pure product as a white solid; m.p. 176.5-178 °C; [α]_D_^25^ = +24.0 (*c* = 0.50, CH_2_Cl_2_); ^1^H-NMR (CDCl_3_): *δ* 7.89 (s, 1H, NH), 7.38-7.33 (m, 3 H, ArH), 7.26-7.13 (m, 5H, ArH), 5.04 (d, *J* = 11.70 Hz, 1H, CH), 4.26 (d, *J* = 11.70 Hz, 1H, CH), 4.03-3.93(m, 4H, 2×CH_2_), 2.38 (s, 3H, CH_3_), 1.02-0.97 (m, 6H, 2×CH_3_); ^13^C-NMR (CDCl_3_): *δ* 167.9, 167.8, 141.3, 134.3, 128.5, 128.2, 128.0, 126.7, 126.6, 123.7, 120.8, 118.7, 116.3, 110.4, 76.6, 61.3, 61.2, 58.3, 42.6, 21.5, 13.5, 13.6. HPLC analysis: t_r_ (minor) = 13.28 min, t_r_ (major) = 16.45 min, 47% ee.

#### 3.4.9. (S)-Ethyl 2-ethoxycarbonyl-3-[3-(5-chloroindolyl)]-3-phenylpropanoate

Prepared according to the general procedure from diethyl benzylidenemalonate and 5-chloroindole to provide the pure product as a white solid; m.p. 190-192 °C; [α]_D_^25^ = -6.0 (*c* = 0.20, CH_2_Cl_2_); ^1^H-NMR (CDCl_3_): *δ* 8.03 (s, 1H, NH), 7.51 (d, *J* = 1.80 Hz, 1H, ArH), 7.36-7.06 (m, 7H, ArH), 6.91 (d, *J* = 2.49 Hz, 1H, ArH), 5.00 (d, *J* = 12.0 Hz, 1H, CH), 1.03-0.98 (m, 6 H, 2×CH_3_); ^13^C-NMR (CDCl_3_): *δ* 167.9, 167.8, 141.1, 134.9, 128.6, 128.5, 128.1, 127.1, 125.4, 122.3, 122.1, 116.8, 113.1, 112.6, 77.4, 61.6, 61.5, 58.6, 42.7, 13.9. HPLC analysis: t_r_ (minor) = 14.88 min, t_r_ (major) = 20.25 min, 45% ee.

#### 3.4.10. (S)-Ethyl 2-ethoxycarbonyl-3-[3-(6-chloroindolyl)]-3-phenylpropanoate

Prepared according to the general procedure from diethyl benzylidenemalonate and 6-chloroindole to provide the pure product as a white solid; m.p. 203-205 °C; [α]_D_^25^ = +20.0 (*c* = 0.25, CH_2_Cl_2_); ^1^H-NMR (CDCl_3_): *δ* 8.02 (s, 1H, NH), 7.42 (d, *J* = 8.40 Hz, 1H, ArH), 7.35-7.15 (m, 7H, ArH), 5.02 (d, *J* = 11.70 Hz, 1H, CH), 4.25 (d, *J* = 12.0 Hz, 1H, CH), 4.04-3.94 (m, 4 H, 2×CH_2_), 1.00 (t, *J* = 7.20 Hz, 2×CH_3_); ^1^^3^C-NMR (CDCl_3_): *δ* 167.9, 167.7, 141.1, 136.5, 128.4, 128.2, 128.1, 126.9, 125.3, 121.5, 120.3, 120.2, 117.2, 110.9, 77.4, 61.5, 61.4, 58.3, 42.7, 13.7. HPLC analysis: t_r_(minor) = 14.93 min, t_r_ (major) = 17.95 min, 10% ee.

## 4. Conclusions

In summary, a series of novel *C_2_*-symmetric fluoren-9-ylidene malonate-derived bis(oxazoline) ligands were synthesized in good yields for the first time from diethyl fluoren-9-ylidene malonate and chiral amino alcohols. Their application in the asymmetric catalytic Friedel-Crafts reaction of indoles and alkylidene malonates was examined. The copper complex of ligand **1d** bearing a phenyl group showed moderate to good enantioselectivity. Further experiments to extend the scope of use of these catalysts are currently in progress in our laboratory.
